# Temporal and spatial patterns of γH2AX signaling in different human cells after exposure to X-rays and UV-C light

**DOI:** 10.1007/s00411-026-01220-z

**Published:** 2026-05-22

**Authors:** Isabella Guardamagna, Leonardo Lonati, Ombretta Iaria, Alice Mentana, Daniele Parodi, Giulia Peterlin, Rossella Semerano, Cecilia Riani, Andrea Previtali, Anna Tricarico, Paola Tabarelli de Fatis, Giovanni Battista Ivaldi, Paola Perucca, Ornella Cazzalini, Giorgio Baiocco

**Affiliations:** 1 Laboratory of Radiation Biophysics and Radiobiology, A. Volta, Department of Physics, University of Pavia, 27100 Pavia, Italy; 2 Unit of Immunology and General Pathology, Department of Molecular Medicine, University of Pavia, 27100 Pavia, Italy; 3 Unit of Medical Physics, Istituti Clinici Scientifici Maugeri IRCCS, 27100 Pavia, Italy; 4 Unit of Radiation Oncology, Istituti Clinici Scientifici Maugeri IRCCS, 27100 Pavia, Italy

**Keywords:** γH2AX, Ultraviolet and ionizing radiation, DNA damage response, UV-C

## Abstract

The phosphorylation of H2AX at serine 139 (γH2AX) is a marker of DNA damage response, well characterized after ionizing radiation (IR) exposure. In this work we compared γH2AX activation in different human cell lines exposed to X-rays, up to 5 Gy, or UV-C light (below ionization threshold), up to 40 J/m^2^. We selected HeLa, Caco-2 and HaCaT cells as models with different origins and radiosensitivity, conventionally screened with the clonogenic assay. We integrated data from fluorescence microscopy and flow cytometry. Results highlight that γH2AX spatial pattern is determined by the genotoxic agent: X-rays induce discrete, countable foci (associated with DNA double-strand breaks), while UV-C leads to pan-nuclear signal distributions. Instead, dose-response curves are strongly dependent on the cell-line, showing linear or saturating trends, not attributable to the readout technique. After 5 Gy X-rays, γH2AX signal peaks within 1 hour. The residual intensity at 24 hours is compatible with the control for all cell lines; however, cells show different clonogenic survival fractions at 7 days. The kinetics of γH2AX positive cells following 20 J/m^2^ UV-C exposure is completely different, with a slower accumulation and persistent signaling from viable cells (not restricted to the S-phase) up to 24 hours, but with a loss of clonogenic potential in the long-term. These findings emphasize that γH2AX marker may be considered as a functional sensor of DNA damage response rather than a marker of specific DNA lesions, and that both radiation quality and cellular context must be considered for its correct interpretation.

**Supplementary Information:**

The online version contains supplementary material available at 10.1007/s00411-026-01220-z.

## Introduction

DNA is the fundamental biological molecule engaged in the perpetuation of life and is intrinsically susceptible to modification and damage by endogenous or exogenous agents. Exposure to electromagnetic (EM) radiation is a common event for humans: the natural EM radiation environment includes both ionizing radiation (IR) and ultraviolet radiation (UV), the latter being below the ionization threshold. Exposure to X-rays is also frequent for medical applications.

UV radiation is divided into three classes based on the wavelength range: UV-C (190-290 nm), UV-B (290-320 nm) and UV-A (320-400 nm) (Diffey 2002). UV-C and UV-B radiation act directly on the DNA causing the formation of photoproducts; the most frequent are pyrimidine dimers (CPDs), in which two adjacent pyrimidines are covalently bound forming a ring structure, and the pyrimidine (6-4) pyrimidone (6-4 photoproduct, 6-4PP), derived from the covalent bond between the C^6^ of a pyrimidine and the C^4^ of the adjacent pyrimidine (Lo et al. 2005). These photoproducts cause a distortion of the DNA helix that can lead to replication errors. The primary source of UV radiation is the sun, whose spectrum reaching Earth is composed of approximately 95% UV-A and 5% UV-B: UV-C and most of UV-B are indeed removed from extraterrestrial radiation by the atmosphere, particularly by the stratosphere (IARC Working Group on the Evaluation of Carcinogenic Risks to Humans 2012).

In the EM spectrum, IR is classically divided in X- and γ- rays based on photon origin and energy (Desouky et al. 2015). These are referred to as indirectly ionizing radiation qualities, because secondary electrons (charged particles) induce ionization events in the biological target. DNA damage caused by IR includes base modifications, single-strand breaks (SSBs) or double-strand (DSBs) (Hutchinson 1985).

UV can lead to the formation of SSBs through the action of reactive oxygen species (ROS) and during DNA repair via nucleotide or base excision (Barnes et al. 2010). At difference with X-rays, UV photons are not able to directly induce DSBs: however, these can occur because of pyrimidine dimers and other modifications distorting the DNA strands, followed by inefficient repair inducing DSBs (Roy 2017). Due to the presence of unrepaired DNA damage, the replication fork can also collapse.

The phosphorylation of H2AX at serine 139 (γH2AX) is a well-established biomarker for DNA damage, particularly following DNA DSBs. After exposure to IR, the γH2AX signal analyzed via fluorescence microscopy is characterized by the appearance of discrete bright spots, referred to as radiation-induced foci (RIF) (Firsanov et al. 2011). For X-rays, the resulting pattern of DNA damage sites and localization of foci is sparsely distributed in the nucleus. Starting from the sites of DNA damage, the H2AX phosphorylation process itself is subject to amplification, and it expands in a ~Mbp (mega base-pairs) genomic region. The γH2AX foci number can be scored by image analysis: it reaches a maximum within 15 – 30 minutes after exposure to IR and then decreases as de-phosphorylation takes place when DNA repair is completed (see, *e.g.,* Mariotti et al. 2013). The maximal number of foci can be counted and correlated with the expected number of initially induced DSBs. For X-rays, observed deviations from a 1:1 DSBs: γH2AX foci correspondence can be understood when specific features of the microscopy technique (as *e.g.* 2D vs. confocal, image resolution), as well as the biological signal amplification are considered (Barbieri et al. 2019). On the contrary, this correspondence does not hold when cells are exposed to densely ionizing radiation (e.g. charged particles), as foci tend to enlarge, merge, and correspond to sites of complex DNA damage, where a single focus may include multiple DSBs (Hagiwara et al. 2017). When scored as a function of dose, foci number generally shows saturating behavior, due to technical and biological limitations, which poses some challenges for its use as a marker for biodosimetry.

Though in absence of direct induction of DSBs, H2AX phosphorylation is measured also after UV exposure. In such case, when analyzed by fluorescence microscopy, the γH2AX signal shows a pan-nuclear distribution (Marti et al. 2006) and discrete foci cannot be resolved, at difference with IR exposures. Also, a different temporal dynamics and intensity can be expected for the signal, because of the different underlying nature of DNA damage and the consequent activation of repair pathways. From a molecular point of view, the activation by phosphorylation of H2AX at the serine 139 can be mediated by ATM/ATR/DNA-PK kinases (Hanasoge and Ljungman 2007).

It is well known that IR activates γH2AX signaling depending on cell-cycle phases, involving homologous recombination in S- and G2- phases, while non-homologous end-joining is the main mechanism in G1-phase (Mao et al. 2008). In the UV case, the nucleotide excision repair is the main pathway involved in all cell-cycle phases (Kciuk et al. 2020).

In this study, we aimed at investigating the induction pattern, intensity, and persistence of the γH2AX signal in response to X-rays, and UV-C light at the threshold of ionization. We compared the response of three human cell lines with a different radiosensitivity to IR, varying the level of exposure (quantified in Gy for X-rays and J/m^2^ for UV-C) and assessing the signal temporal dynamics after exposure. We present results from fluorescence microscopy (also providing information on the spatial distribution of the signal in cell nuclei), flow cytometry (for γH2AX signal intensity and cell viability), and finally clonogenic assays, to investigate the relationship between γH2AX persistence after exposure and long-term cell survival.

## Materials and methods

### Cell culture

HeLa S3 cells (cervical cancer cell line, ATCC), Caco-2 cells (colorectal adenocarcinoma, ATCC) and HaCaT cells (immortalized keratinocyte cell line, IZSLER) were cultured in Dulbecco’s Modified Eagle’s medium (DMEM, EuroClone) supplemented with 10% fetal bovine serum (FBS, EuroClone), 2 mM L-glutamine (EuroClone), 100 U/ml penicillin, 100 mg/ml streptomycin (EuroClone) at 37 °C in a humidified atmosphere with 5% CO_2_. Cells were passaged around 75-80% of confluence.

### X-ray and UV-C irradiations

Irradiations with X-rays were performed at the Radiotherapy Department of Istituto di Ricovero e Cura a Carattere Scientifico (IRCCS) S. Maugeri (Pavia, Italy) with a linear accelerator routinely used for radiotherapy treatments. Cells were exposed to different doses: 0 (Sham), 0.5, 1, 2, 5 Gy, as specifically indicated for each experiment. Irradiations were performed as previously described in detail (Babini et al. 2018). Cell exposures to UV-C were performed at the Molecular Medicine Department (University of Pavia) using a lamp (Philips TUV-9, Philips) emitting mainly at 254 nm, with exposure levels of 10, 20, 40 J/m^2^ measured with a DCRX radiometer (Spectronics), as specifically indicated for each experiment. For the sake of simplicity, the word “*dose*” is used in this work to indicate both the absorbed dose for IR (Gy) and exposure levels (J/m^2^).

### Clonogenic survival assay

HeLa, Caco-2 and HaCaT cells were seeded in 6-well plates, at a density of 250 cells per well in control samples or 500 cells per well in irradiated conditions. Twenty-four hours after seeding, samples were irradiated with X-rays and cells were then incubated at 37 °C in a humidified atmosphere with 5% CO_2_ for 7 days; colonies were then fixed and stained with a solution containing 1% Crystal Violet (Sigma-Aldritch), and counted with a colony counter (SC6Plus, Stuart), following protocols as in (Guardamagna et al. 2021) consistent with the original standardized protocol described by (Franken et al. 2006). As conventionally done in clonogenic studies, survival fraction data are given as number of colonies scored divided by number of cells seeded, normalized to the plating efficiency measured in control conditions. Methods and results for the clonogenic survival assay after UV-C exposures are reported as Supplementary Material to this work.

### γH2AX immunostaining for fluorescence microscopy

HeLa, Caco-2 and HaCaT cells were seeded at 2×10^5^ cells per 35 mm Petri dish containing a 22×22 mm glass coverslip. 24 hours after seeding, cells were irradiated with UV-C or X-rays, as previously described. Thirty minutes post-irradiation, cells were washed with PBS, fixed with 4% formaldehyde for 5 minutes, and permeabilized in 70% ethanol at −20 °C for at least 20 minutes. Samples were subsequently washed twice with cold PBS 1X and incubated in blocking solution (PBS 1X with 0.2% Tween-20 and 5% BSA). After blocking, samples were incubated with the primary anti-γH2AX antibody (1:400 dilution, Cell Signaling Technologies, RRID: AB_2118011) for 1 hour, followed by incubation with Alexa Fluor 555-conjugated anti-rabbit secondary antibody (1:200 dilution, ThermoFisher Scientific, RRID: AB_1500773) for 30 minutes. Nuclear DNA was counterstained with Hoechst 33342 dye (1:6000 dilution, Abnova) for 10 minutes at room temperature under gentle agitation, followed by washing in PBS 1X. Coverslips were mounted using Mowiol (Calbiochem) containing 0.25% 1,4-diazabicyclo-octane (Aldrich) as an antifading agent. Images were acquired using a 100X magnification objective on a fluorescence microscope (Olympus BX51, Olympus) equipped with a digital CCD camera (Retiga-2000R, QImaging). For each experimental condition, 7 randomly selected fields were imaged, with approximately 10 nuclei per field, with standardized acquisition setup including fixed exposure times, leading to absolute fluorescence intensities comparable between samples. Quantitative analysis of γH2AX fluorescence intensity was performed using the open-source software ImageJ v.1.52a (NIH), and expressed as MFI (*mean fluorescence intensity*) values per cell, after background correction. Results for investigated conditions are given as the relative fractional variation of this quantity with respect to the control: $\Delta frac MFI = \left(MF{I}_{condition }- MF{I}_{control}\right)/MF{I}_{control}$  

### Monoparametric γH2AX flow-cytometry analysis

HeLa, Caco-2 and HaCaT cells were seeded at 5x10^5^ cells per 65 mm Petri dish (for UV irradiated samples) or T25 flask (for X-rays irradiated samples) and, 24 h after seeding, samples were irradiated with UV-C or X-rays. Cells were then incubated at 37 °C in a humidified atmosphere with 5% CO_2_. Cells were collected by trypsinization at defined time points and then fixed with 4% (w/v) paraformaldehyde for 5 min and permeabilized with 70% v/v ethanol in 0.9% w/v NaCl. Fixed and permeabilized cells were incubated in blocking solution (PBS 1X, 0.2% Tween-20 containing 5% BSA) and subsequently incubated with the anti-γH2AX primary antibody (1:200 dilution, Cell Signaling Technology, RRID: AB_2118011) for 1 h, and then with the secondary anti-rabbit antibody conjugated with Dylight 488 (1:200 dilution, Cell Signaling Technology, RRID:AB_10679405) for 30 min. DNA staining was obtained using the FxCycle™ Violet Stain kit (ThermoFisher Scientific). An Attune NxT Acoustic Focusing flow cytometer (ThermoFisher Scientific) was used for the analysis of stained samples. All the collected data were analyzed using the Attune NxT software v 4.2.1627.1. At least 30 000 events per sample were acquired and the MFI values (median fluorescence intensity) were derived from the monoparametric spectra of the γH2AX signal after gating on the DNA signal for singlet cells. Results for investigated conditions are given as the relative fractional variation of this quantity with respect to the control: $\Delta \mathrm{f}\mathrm{r}\mathrm{a}\mathrm{c} MFI = ({MFI}_{\mathrm{c}\mathrm{o}\mathrm{n}\mathrm{d}\mathrm{i}\mathrm{t}\mathrm{i}\mathrm{o}\mathrm{n} }- {MFI}_{\mathrm{c}\mathrm{o}\mathrm{n}\mathrm{t}\mathrm{r}\mathrm{o}\mathrm{l}})/{MFI}_{\mathrm{c}\mathrm{o}\mathrm{n}\mathrm{t}\mathrm{r}\mathrm{o}\mathrm{l}}$

Methods and results for cell-cycle phase-resolved data for γH2AX activation are reported as Supplementary Material to this work.

### Dual parameter Live/Dead and γH2AX flow-cytometry analysis

HeLa, Caco-2, and HaCaT cells were seeded at 5 × 10^5^ cells per 65 mm Petri dish. After 24 hours of incubation, samples were irradiated with 20 J/m^2^ UV-C, as previously described. Following irradiation, cells were incubated at 37 °C in a humidified atmosphere with 5% CO₂. At defined time points, cells were harvested by trypsinization and centrifuged at 740 × g (IEC CL31R multispeed centrifuge, ThermoFisher Scientific). Supernatants were discarded, and cell pellets were re-suspended in a Live/Dead staining solution (Live/Dead Fixable Dead Cell Stain Kits, Molecular Probes), according to the manufacturer’s instructions. Cells were subsequently fixed in 4% paraformaldehyde and stored in 70% v/v ethanol in 0.9% w/v NaCl at −20 °C. For γH2AX detection, cells were centrifuged, re-suspended in blocking solution (PBS with 0.2% Tween-20 and 5% BSA), and incubated at room temperature for 30 minutes. Cells were then incubated with the primary anti-γH2AX antibody (1:200 dilution, Cell Signaling Technology, RRID: AB_2118011) for 1 hour, followed by incubation with an Alexa Fluor 488-conjugated anti-rabbit secondary antibody (1:200 dilution, Cell Signaling Technology, RRID: AB_1904025) for 30 minutes. Stained samples were analyzed using an Attune NxT Acoustic Focusing Flow Cytometer (ThermoFisher Scientific). Flow cytometry data were acquired and processed using Attune NxT software version 4.2.1627.1. At least 30 000 events per sample were gated based on physical parameter discrimination, then on viability dye intensity to delineate live (L/D-) and dead (L/D+) populations. The percentage of events and the MFI values of γH2AX were recorded in each gate. Results for investigated conditions are given as the relative fractional variation of this latter quantity with respect to the control: $\Delta \mathrm{f}\mathrm{r}\mathrm{a}\mathrm{c} MFI = ({MFI}_{\mathrm{c}\mathrm{o}\mathrm{n}\mathrm{d}\mathrm{i}\mathrm{t}\mathrm{i}\mathrm{o}\mathrm{n} }- {MFI}_{\mathrm{c}\mathrm{o}\mathrm{n}\mathrm{t}\mathrm{r}\mathrm{o}\mathrm{l}})/{MFI}_{\mathrm{c}\mathrm{o}\mathrm{n}\mathrm{t}\mathrm{r}\mathrm{o}\mathrm{l}}$

### Statistical analysis

For the analysis of clonogenic survival data after X-ray exposure, the linear quadratic model was applied to extract the α and β values for each cell line with the CFAssay package v 1.42.0 in R v 4.3.3 (Braselmann et al. 2015). The package uses Maximum Likelihood and provides a dispersion parameter to assess the goodness of the fit. Data are given as average values among 4 biological replicates ± SEM (standard error of the mean).

For the analysis of fluorescence microscopy images, 7 randomly selected fields were acquired for each experimental condition, with approximately 10 nuclei per field. Data are given as average values among 3 biological replicates ± SEM.

In all flow-cytometry experiments, at least 30000 events were acquired per sample. Data on fluorescence intensity of γH2AX with different techniques (immunofluorescence microscopy and flow cytometry) were analyzed in GraphPad Prism v 10.1.2 and Python v 3.12, using nonlinear regressions or ANOVA and post-hoc multiple comparison tests, as detailed for each dataset.

In particular, for each cell line, dose-response data were fitted separately to candidate models: a linear model, with $\Delta \text{frac MFI} =m\cdot d+q$, or an exponential function allowing for signal saturation, $\Delta \text{frac MFI} = a\cdot (1-{e}^{-b\cdot d})$, where *d* is the dose and *m*, *q, a*, *b* are free parameters. For all cell lines, the kinetics of γH2AX signal activation was reproduced by the analytical function: $\Delta \text{frac MFI} = \mathrm{A}\cdot \left(1-{e}^{-B\cdot t}\right)\cdot {e}^{-C\cdot t}$, where *t* is the post-exposure time, and* A*,* B* and* C* are free-parameters).  

One-way ANOVA and subsequent multiple t-test corrected with Benjamini-Hochberg correction factors, were employed to test statistical significance of observed differences in viability data as function of time and cell lines, and in γH2AX signal as function of time and cell lines, after selecting live or dead cells based on the viability marker. All the experiments were performed in at least 3 independent biological replicates.

## Results

### Clonogenic survival data and application of the linear quadratic model after X-ray exposure

**Fig. 1:**
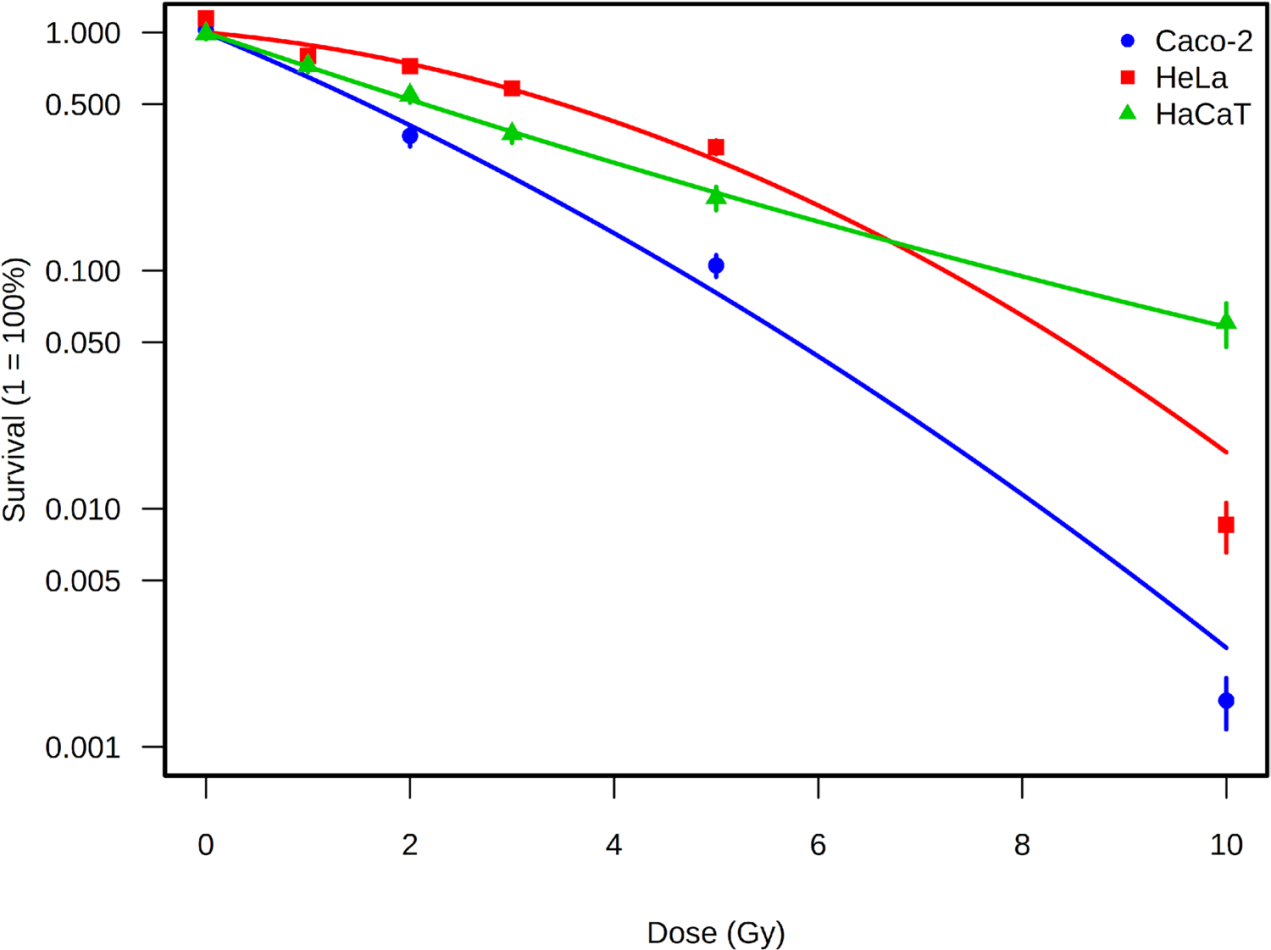
Survival fraction data and best-fit curves obtained with the linear quadratic model for Caco-2 cells (blue dots), HeLa cells (red squares), and HaCaT cells (green triangles), exposed to different doses of X-rays. Data are expressed as means ± SEM of at least 4 replicates

The HeLa, Caco-2 and HaCaT cell lines were chosen after screening of radio-sensitivity/resistance, standardly quantified via the clonogenic survival assay. Figure 1 shows the experimental data on surviving fraction vs. X-ray dose for all cell lines. Data are fitted with the linear quadratic model to extract the parameters α and β reported in Table 1. The survival fraction at 2 Gy (SF2) is also commonly adopted as a quantitative indicator of radioresistance, and the following values and ranking can be extracted from the data analysis: 79.6% (±1.9%) for HeLa; 72.5% (±4.4%) for HaCaT; and 36.8% (±3.6%) for Caco-2. However, given the shape of the surviving fraction curves, it is interesting to notice that HaCaT cells show a higher radioresistance (higher surviving fraction) at the highest 10 Gy dose. A single parameter as SF2 may not be enough to correctly interpret the overall response to X-rays.

**Table 1:** α and β parameters obtained from the linear quadratic model applied to experimental data on survival fraction of X-ray-exposed Caco-2, HeLa and HaCaT cells

Cell-line	α (Gy^-1^)	β (Gy^-2^)	α/β (Gy)	SF2	‍Dispersion parameter
Caco-2	0.412±0.039	0.018±0.005	22.89±6.72	36.8% (±3.6%)	‍4.58
HeLa	0.088±0.031	0.032±0.005	2.75±1.06	79.6% (±1.9%)	‍3.05
HaCaT	0.335±0.021	0.005±0.003	67.00±40.42	72.5% (±4.4%)	‍0.36

Results for cell survival after exposure to UV-C are reported as Supplementary Material to this work.

### Spatial pattern of γH2AX signal after X-ray and UV-C exposures from fluorescence microscopy images

**Fig. 2:**
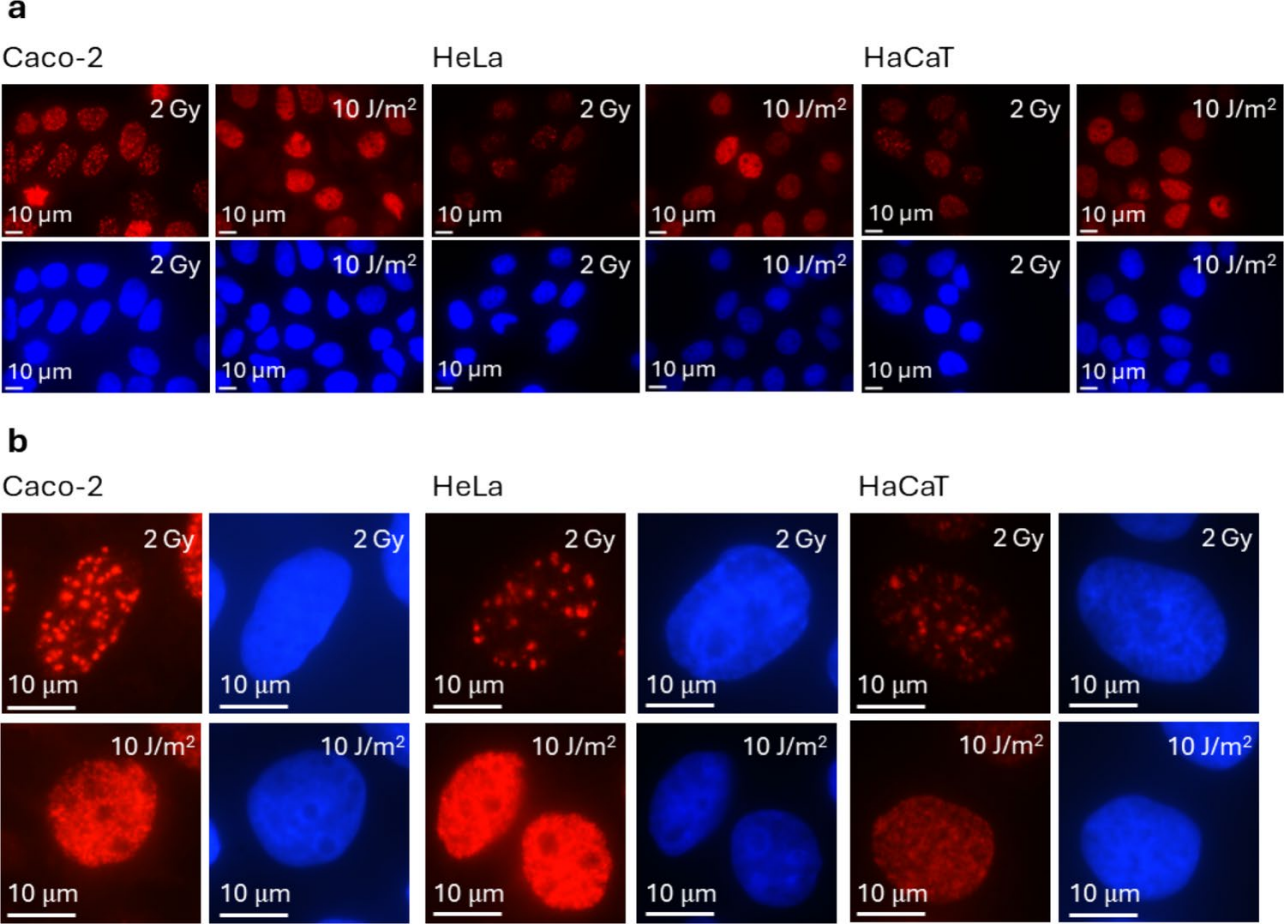
γH2AX activation detected by immunofluorescence. Representative images of HeLa, Caco-2 and HaCaT cells marked with γH2AX in red and Hoechst in blue, after exposure to 2 Gy X-rays or 10 J/m^2^ UV-C. (**a**) Acquisition at 100X magnification, scale bar 10 µm. (**b**) Zoom of representative nuclei, scale bar 10 µm

The activation of γH2AX signaling following irradiation with X-rays (2 Gy) and UV-C (10 J/m^2^) was observed in fluorescence microscopy at 30 minutes after exposure. Figure 2a shows representative images of the three cell lines after irradiation, with Hoechst staining (blue) for cell nuclei and γH2AX signals in red, acquired at 100X magnification. Comparing the response to UV-C and X-rays, a different spatial pattern of the signal is evident, which can be more clearly observed in the magnified images shown in Fig. 2b: discrete γH2AX foci, commonly associated with DNA damage induced by ionizing radiation, appear in nuclei exposed to X-rays, while a pan-nuclear and more homogeneously distributed fluorescence intensity is observed throughout nuclei exposed to UV-C. Based on this finding, it appears evident that the classical scoring of foci number can only be applied to images from samples exposed to X-rays (Figure S1). In what follows, integrated signal intensity per cell is used to compare γH2AX activation after exposure to X-rays and UV-C.

### Dose-response of γH2AX signal after X-ray and UV-C exposures from fluorescence microscopy image analysis

**Fig. 3:**
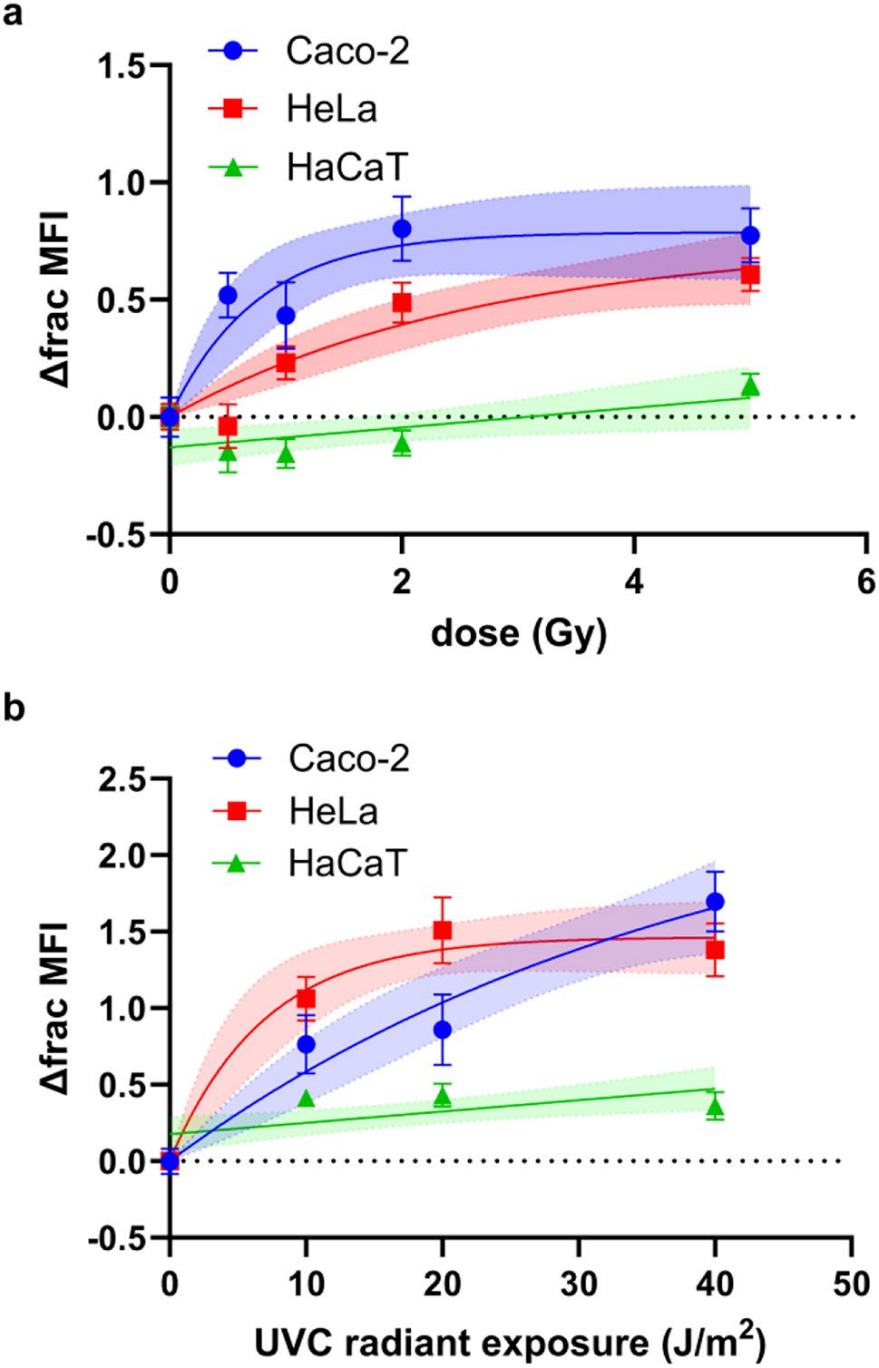
Dose-response to X-ray (**a**) and UV-C (**b**) exposures for Caco-2 (blue dots), HeLa (red squares) and HaCaT (green triangles) cells, measured 30 minutes after irradiation, as relative increase of mean fluorescence intensity per cell with respect to the control (Δfrac MFI). Data are obtained from a minimum of 100 nuclei per samples acquired at 100X magnification, and expressed as mean ± SEM. Analytical best-fit functions are either linear ($\Delta \mathrm{f}\mathrm{r}\mathrm{a}\mathrm{c} MFI =m\cdot d+q$ where *d* is dose of irradiation, either from X-ray or UV-C, and *m* and* q* the free parameters) or exponential ($\Delta \mathrm{f}\mathrm{r}\mathrm{a}\mathrm{c} MFI = a\bullet (1-{e}^{-b\cdot d})$, where *d* is dose of irradiation and *a* and *b* free parameters), allowing for signal saturation, and colored bands represent 95% confidence intervals

The quantification of the relative change in mean fluorescence intensity with respect to the control (Δfrac MFI) from the analysis of microscopy images revealed a differential response of the three cell lines to increasing levels of exposure to X-rays and UV-C. Following exposure to X-rays (Fig. 3a), a net increase in MFI was observed in Caco-2 and HeLa cells, while HaCaT showed a poor signal activation, comparable to the control level. Similarly, exposure to UV-C radiation (Fig. 3b) induced a dose-dependent increase in fluorescence, with a less marked increasing trend in HaCaT cells. To support data interpretation, different best-fit curves were applied: a better agreement was found with either a linear trend or an exponential curve allowing for signal saturation, depending on the cell-line and kind of radiation. Signal saturation is commonly observed in γH2AX activation vs. dose for IR exposure, and it is known to be also affected by technique limitations. Dose-response relationships were therefore also measured from flow-cytometry data, and results are presented in the following. Tables summarizing the trends observed and the best-fit parameters, together with their uncertainties and figures of merits, are included as Supplementary Material (Table S1).

### Dose-response of γH2AX signal after X-ray and UV-C exposures from flow-cytometry analysis

**Fig. 4:**
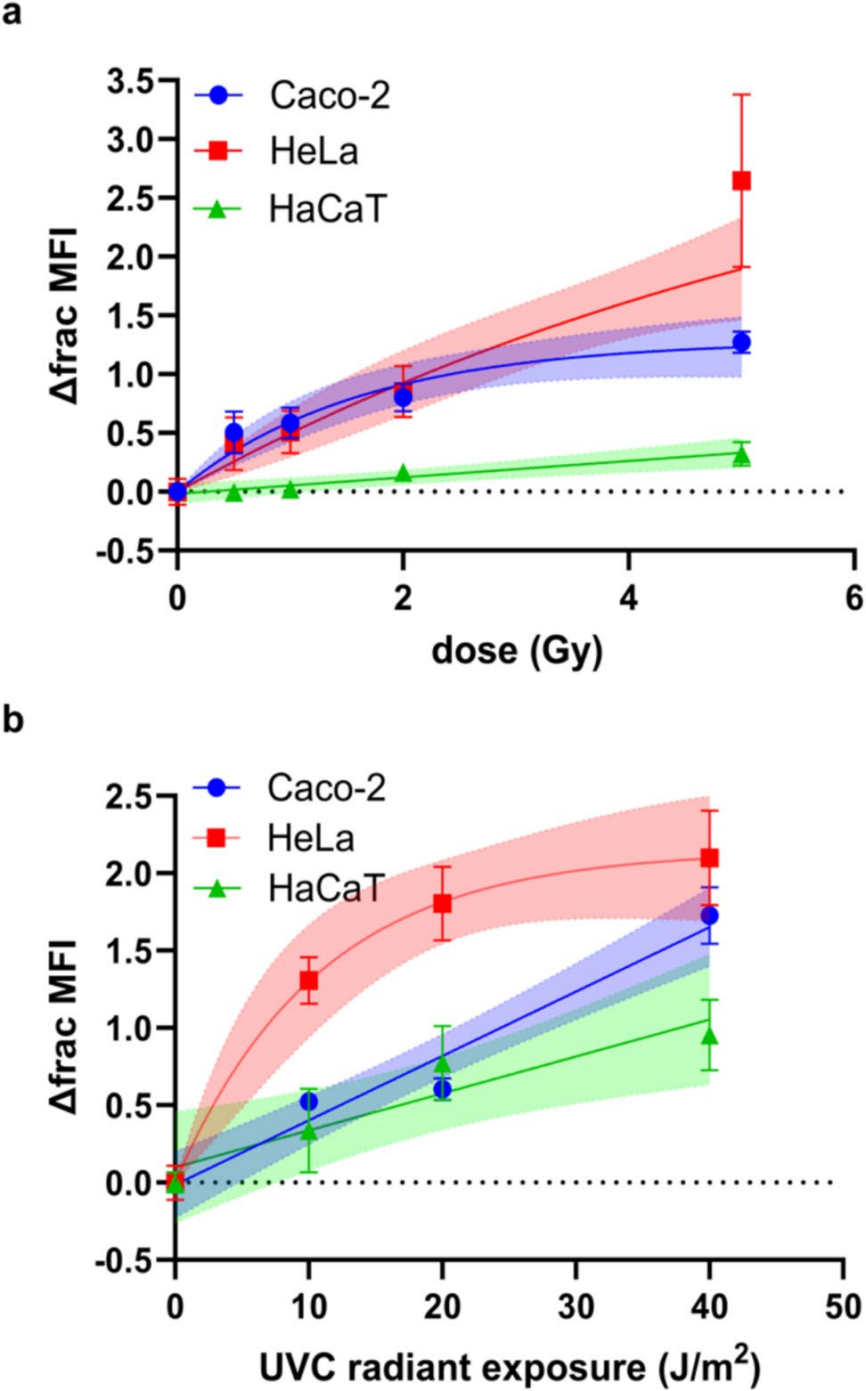
Dose-response to X-ray (**a**) and UV-C (**b**) exposures for Caco-2 (blue dots), HeLa (red squares) and HaCaT (green triangles) cells, measured 30 minutes after irradiation, as relative increase of median fluorescence intensity per cell with respect to the control (Δfrac MFI). Data are obtained in flow cytometry from a minimum of 30000 events per samples and expressed as mean ± SEM from at least 3 biological replicates for each cell line. Analytical best-fit functions are either linear ($\Delta \mathrm{f}\mathrm{r}\mathrm{a}\mathrm{c} MFI =m\cdot d+q$, where *d* is dose of irradiation and *m* and *q* the free parameters) or exponential ($\Delta \mathrm{f}\mathrm{r}\mathrm{a}\mathrm{c} MFI = a\cdot (1-{e}^{-b\bullet d})$, with *a* and *b* free parameters), allowing for signal saturation, and coloured bands represent 95% confidence intervals

To clarify the origin of the non-linear response observed in the microscopy-based quantification of γH2AX fluorescence, a complementary analysis was conducted by means of flow cytometry. The monoparametric distribution of the γH2AX signal revealed a dose-dependent shift in median fluorescence intensity towards higher values in irradiated cells with respect to the control population, confirming increasing levels of γH2AX activation following increasing levels of exposure to X-rays (Fig. 4a) and UV-C (Fig. 4b) radiation. HeLa cells exhibited a clearly distinguishable γH2AX-positive peak even at lower doses, whereas Caco-2 and HaCaT cells displayed a more gradual response. Data are shown in Fig. 4 as relative change in median fluorescence intensity with respect to the control (Δfrac MFI) vs. dose and were fitted to support data interpretation. In most cases, MFI values vs. dose follow a linear behaviour, and signal saturation is required only to reproduce data for X-ray-exposed Caco-2 cells and UV-C exposed HeLa cells. Tables summarizing the trends observed and the best-fit parameters, together with their uncertainties and figures of merits, are included as Supplementary Material (Table S2). Comparing data shown in Figs. 3, 4, it is evident that a higher increase in signal intensity can be measured by means of flow cytometry rather than microscopy. However, the similarity in the trends that can be inferred suggest that the observed non-linearity stems from intrinsic regulatory mechanisms governing γH2AX activation depending on the ​​cell type, rather than from technical limitations.

### Kinetics of the γH2AX signal after X-ray and UV-C exposures from flow-cytometry analysis

**Fig. 5:**
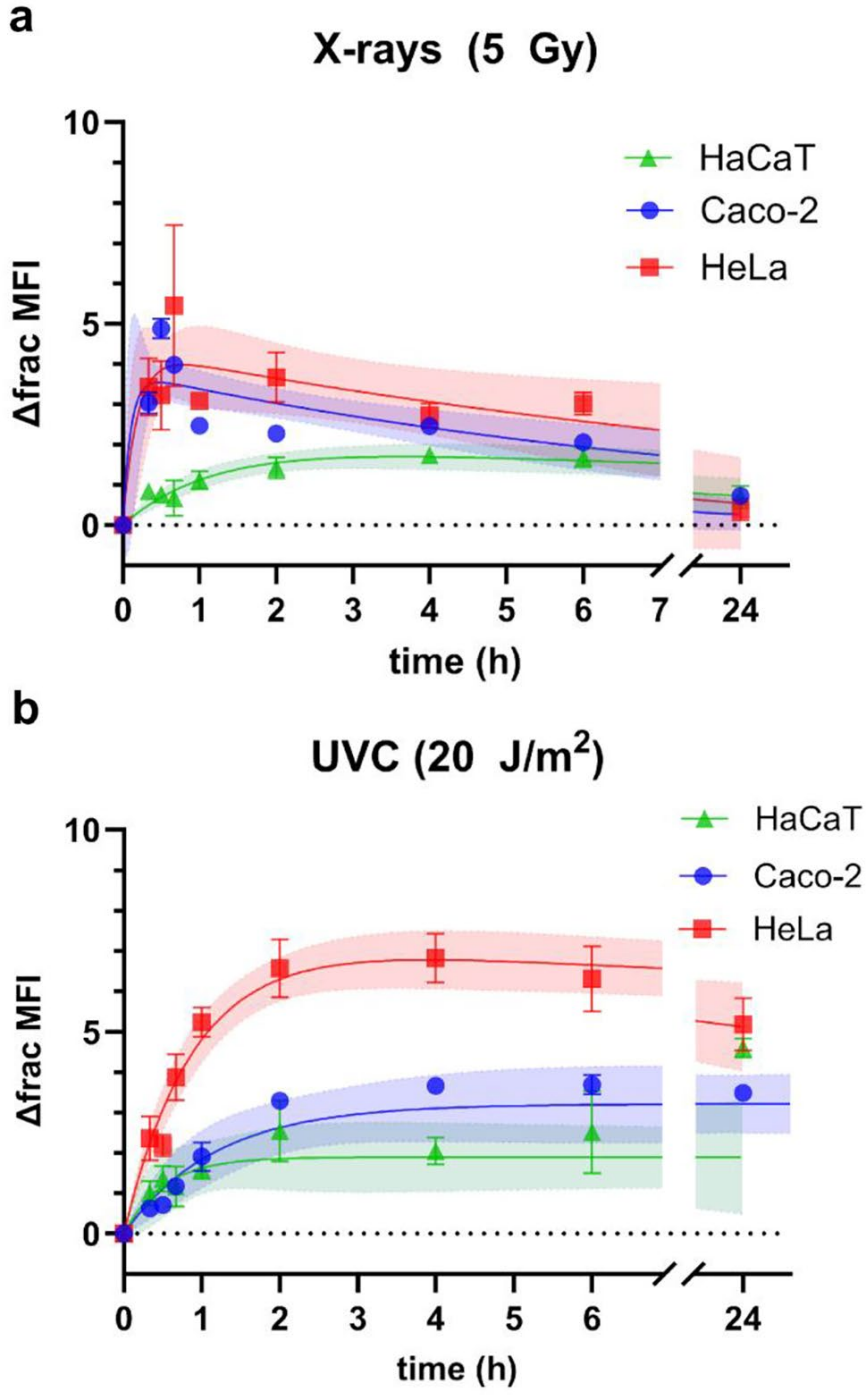
γH2AX signaling kinetics after exposure to 5 Gy X-rays (**a**) or 20 J/m^2^ UV-C (**b**) exposures analyzed for Caco-2 (blue dots), HeLa (red squares) and HaCaT (green triangles) cells. Data are measured as median fluorescent intensity from the flow-cytometry analysis and expressed as Δfrac MFI ± SEM from at least 3 biological replicates for each cell line. Best fit analytical functions have the following expression: $\Delta \mathrm{f}\mathrm{r}\mathrm{a}\mathrm{c} MFI = \mathrm{A}\cdot \left(1-{e}^{-B\cdot t}\right)\cdot {e}^{-C\cdot t}$ (where *t* is the post-exposure time, and *A, B* and *C* are free-parameters) and support data interpretation

To assess the temporal dynamics of the γH2AX activation following irradiation with X-rays and UV-C we selected two reference exposure levels and measured the fluorescence intensity by means of flow cytometry at different time points after exposure. Based on the findings reported in the dose-response curves of Fig. 4, a dose of 5 Gy for X-rays and an exposure level of 20 J/m^2^ for UV-C were chosen, to be sure that the relative increase of the γH2AX signal at 30 minutes would be significantly higher than the control condition for all cell lines (including HaCaT cells, always showing a weak re

sponse). Results for the signal kinetics are reported in Fig. 5. After treatment with 5 Gy X-rays, all cell lines exhibited a quick and marked increase in γH2AX signal (Fig. 5a): the signal reaches a maximal level within 1 hour post irradiation for HeLa and Caco-2 cells, and the magnitude of the effect is higher when compared to HaCaT. After the maximum, the signal starts decreasing for HeLa and Caco-2 cells, while some persistence is observed for HaCaT. At 24 hours after irradiation, however, the signal gets back to basal level for all cell lines.

Exposure to 20 J/m^2^ led to elevated γH2AX levels in all cell lines, albeit with distinct kinetics compared to IR (Fig. 5b): the increase after exposure is less steep, seemingly reaching a plateau level in ~2 hours. At longer times, all cell lines showed a sustained γH2AX signal, with different magnitudes of the effect, suggesting differences in damage processing or repair pathway engagement. At the 24-hour time point, residual γH2AX levels remained elevated, suggesting either persistent DNA damage and delayed repair mechanisms, or DNA fragmentation associated with cell death. Cell-cycle phase analysis (Figures S2-S3) showed that the distribution of cells across different phases did not substantially influence γH2AX activation, indicating that the observed kinetics are largely independent of cell-cycle status.

For all datasets, best-fit curves of the type were applied to support data interpretation. Tables summarizing best-fit parameters, together with their uncertainties and figures of merits, are included as Supplementary Material (Table S3).

### Dual parameter γH2AX and Live/Dead after UV-C exposures from flow-cytometry analysis

**Fig. 6:**
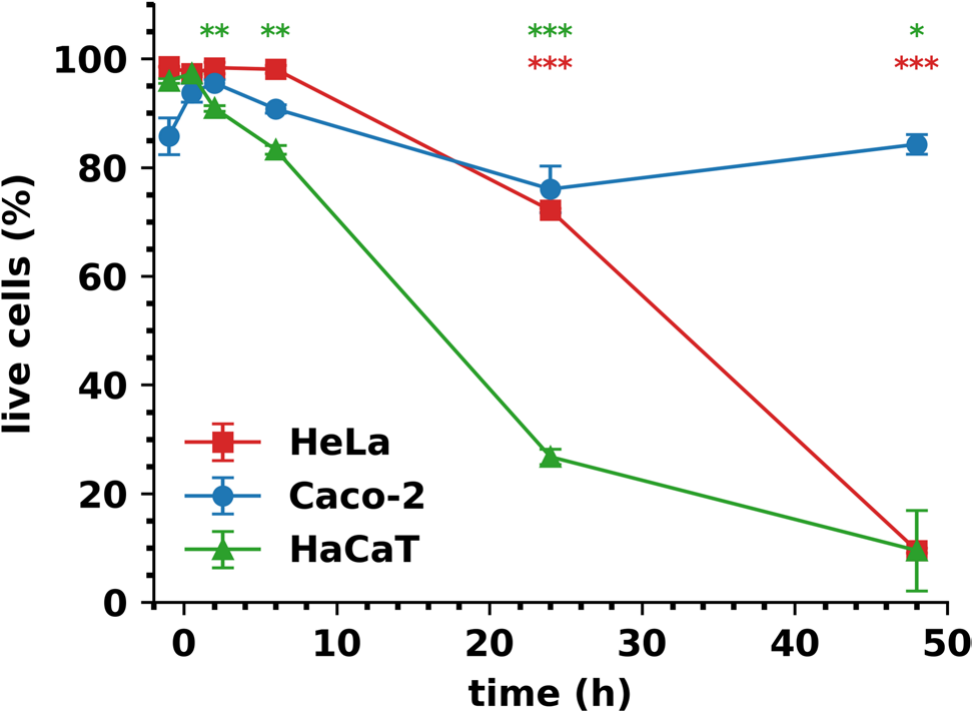
Percentage of viability vs. time after exposure to 20 J/m^2^ UV-C for Caco-2 (blue dots), HaCaT (green triangles) and HeLa (red squares). Data are given as mean *±* SEM for at least 3 biological replicates. Significance is obtained from multiple t-test corrected with Benjamini-Hochberg correction, after ANOVA test: * p-value<0.05, ** p-value<0.01, *** p-value<0.005

**Fig. 7:**
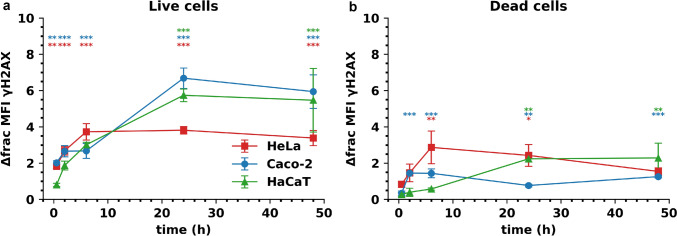
Results from the dual parameter γH2AX and Live/Dead flow-cytometry analysis on the γH2AX signal intensity (Δfrac MFI) vs. time, after exposure to 20 J/m^2^ UV-C for viable (**a**) and dead (**b**) Caco-2 (blue dots), HaCaT (green triangles) and HeLa (red squares) cells. Data are given as mean *±* SEM for at least 3 biological replicates. Significance is obtained from multiple t-test corrected with Benjamini-Hochberg correction, after ANOVA test: * p-value<0.05, ** p-value<0.01, *** p-value<0.005

To investigate the origin of the persistence of elevated γH2AX levels at late time points following UV-C exposure, we performed a dual-parameter flow-cytometry analysis, combining γH2AX staining with a Live/Dead viability marker. Cells were exposed to 20 J/m^2^ and analyzed as a function of post-irradiation time. This approach allowed us to discriminate between γH2AX-positive cells that maintained viability from those undergoing cell death. Results are reported in Fig. 6, where cell viability is defined as the percentage of live cells normalized with respect to the unirradiated condition. The analysis revealed a net decrease in cell viability as a function of time for HaCaT, leading to 26.8±2.4% of viable cells at 24 h, while HeLa and Caco-2 cells, at the same time-point, have viable fractions of 72.1±0.6% and of 76.0±7.4%, respectively. Caco-2 viability did not appear to be further affected even at the latest 48 h time-point, while live cells dropped to less than 10% for both HaCaT and HeLa within the same timeframe (9.5±7.8% and 9.6±1.2% respectively). However, survival assays following UV-C exposure revealed a markedly reduced clonogenic survival for all cell lines (below 10^-3^ surviving fraction at the same exposure level, see Figure S4 in Supplementary Material). Moreover, from the same dataset, Δfrac MFI values for the γH2AX signal can be obtained gating on live cells or on dead cells only: in live cells, the signal keeps increasing for all cell lines along time, reaching a plateau at 24 - 48h (Fig. 7a). Also, cells that are recognized as dead show an increase in the fold change of the γH2AX signal along time, but with a lower signal amplification with respect to the control (Fig. 7b).

## Discussion

This work aimed at characterizing γH2AX as a sensor of DNA damage and DNA damage response after exposure to X-rays and UV-C. Measurements were conducted comparing three human cell lines with different radiosensitivity to IR, screened with the clonogenic assay.

Results obtained through microscopy confirm that X-ray exposure induces a classical pattern of discrete γH2AX nuclear foci, as expected in the case of a sparse distribution of DNA DSBs (Rogakou et al. 1998). UV-C exposures instead result in a pan-nuclear γH2AX activation, likely reflecting ATR-mediated signaling in response to DNA damage processing, rather than a direct consequence of photolesions such as CPDs or double-strand breaks (Marti et al. 2006; Hanasoge and Ljungman 2007). γH2AX foci counting is therefore not applicable to compare the response of the same cell lines to these two stressors, and integrated fluorescence in cell nuclei shall be quantified. This can be done via microscopy image analysis, but flow cytometry offers the chance of higher throughput measurements. In this work, the combination of the two techniques is adopted, obtaining compatible and complementary results: we showed that the intensity and γH2AX activation varies significantly depending on the type of EM radiation, dose/exposure level, and intrinsic properties of each cell line. In particular, when signal saturation is observed as a function of increasing dose, this is confirmed to be a specific feature of the cell line response to the stressor under study, and not merely related to technique limitations. However, if γH2AX activation is used with the purpose of comparing DNA damage induction (which is more appropriate for the DSB direct induction by IR), comparisons among cell lines should be done with caution and only in the dose range where the signal remains linear with the dose (Tanaka et al. 2007).

The kinetics of γH2AX signal accumulation and resolution after exposure further emphasized cell-line and radiation-type specific differences. After X-ray exposure, γH2AX peaked at ~30 minutes for Caco-2 and HeLa (with Δfrac MFI up to ~5), while a maximum was reached in ~2 hours for HaCaT, with a lower intensity level (Δfrac MFI up to ~2). For all cell lines, the signal intensity declined (within few hours for Caco- 2 and HeLa), going back to levels compatible with the control at 24 hours. γH2AX induction following UV-C irradiation displayed a slower and more sustained profile. This observation is in line with prior studies reporting γH2AX persistence up to 12–24 hours post-UV exposure, particularly when DNA repair is inefficient or replication forks remain stalled (Hanasoge and Ljungman 2007). The proposed live/dead co-staining approach confirmed however that the γH2AX signal at late time points was not limited to apoptotic or necrotic cells, but was mainly detectable in viable cells: at 24 hours post-exposure, 72.1±0.6% of HeLa and 76.0±7.4% of Caco-2 cells irradiated with 20 J/m^2^ UV-C are marked as living and with a high γH2AX signal (Δfrac MFI ~4 and ~8, respectively). This suggests that γH2AX can be maintained in cells experiencing chronic genotoxic stress rather than only reflecting terminal damage.

Overall, our findings align with the interpretation that γH2AX is not a direct marker of primary UV-induced photolesions such as CPDs, but rather reflects downstream signaling events associated with DNA damage processing (Hanasoge and Ljungman 2007). Several works (Ward and Chen 2001; Ward et al. 2004; Tanaka et al. 2007) reported high signals of γH2AX in UV-irradiated cells in S-phase. Indeed, SSBs formed during synthesis can be converted to DSBs with an unchecked replication. However, other studies (de Feraudy et al. 2010) have challenged the notion that S-phase cells are the most vulnerable population to DNA damage, showing that only a minority of γH2AX foci in S-phase after UV irradiation represent DSBs. Dhuppar et al. also studied cell cycle-dependent DNA damage response to UV with imaging techniques, showing that H2AX phosphorylation accumulates at replication sites, without co-localization with photoproducts/DNA damage sites (Dhuppar et al. 2020). This slows down the replication and finally lead to a block of replication forks. In this context, our phase-resolved flow-cytometry data (Supplementary Material, Figures S2-3) showed instead higher γH2AX signal amplification in G1-phase cells, particularly following UV-C exposure. This suggests that H2AX phosphorylation can be robustly activated also in non-replicating cells (Guardamagna et al. 2020), possibly in response to DNA damage processing during nucleotide excision repair or ATR-mediated signaling triggered by persistent photolesions. These results underscore the need for caution when interpreting γH2AX as a phase-specific marker of replication-associated stress.

Finally, to evaluate whether persistent γH2AX activation correlates with long-term cellular outcomes, we considered clonogenic survival as a functional readout. Previous studies integrating results for a variety of cell lines (Klokov et al. 2006) suggested that a high level of residual γH2AX signal24 hours after exposure to IR is an indicator of higher radiosensitivity (quantified as a smaller surviving fraction at 2 Gy). Flow-cytometry data for the signal kinetics after 5 Gy X-rays indicate that the three lines under investigation have almost no measurable residual γH2AX activation at 24 hours, though showing a different level of radiosensitivity in terms of maintained clonogenic ability. For UV-C exposures, the sustained γH2AX expression measured in viable cells up to 48 hours from the irradiation, coupled with results from the Live/Dead staining indicating an increase of the dead cell percentage over the same time frame, seems coherent with the observed strong reduction in colony-forming ability following irradiation (Halicka et al. 2005).

## Conclusions

By exposing cell lines with different origin and radiosensitivity (HeLa, Caco-2, and HaCaT) to X-rays and UV-C, we demonstrate that γH2AX activation is not strictly determined by the nature of the genotoxic agent (ionizing vs. non-ionizing radiation) and its potential to directly induce DNA DSBs. Instead, γH2AX signalling, both in terms of dose-response and kinetics, is tightly linked to cellular phenotype and to the efficiency and integrity of DNA damage response pathways. Although it remains a highly sensitive and quantifiable biomarker of radiation exposure, its interpretation requires careful contextualization within the broader biological framework of the system under investigation. By integrating data obtained through multiple complementary techniques, this study provides a more comprehensive characterization of γH2AX as a sensor of genotoxic stress, laying the groundwork for future targeted investigations aimed at elucidating the molecular pathways governing its activation.

## Supplementary Information

Below is the link to the electronic supplementary material.

Supplementary file 1.

## Data Availability

The experimental datasets analyzed during the current study are available from the corresponding author on reasonable request.

## References

[CR1] Babini G, Morini J, Barbieri S et al (2018) A Co-culture Method to Investigate the Crosstalk Between X-ray Irradiated Caco-2 Cells and PBMC. JoVE. 10.3791/56908 10.3791/56908 PMC5912320 29443050

[CR2] Barbieri S, Babini G, Morini J et al (2019) Predicting DNA damage foci and their experimental readout with 2D microscopy: a unified approach applied to photon and neutron exposures. Sci Rep 9:14019. 10.1038/s41598-019-50408-5 31570741 10.1038/s41598-019-50408-5 PMC6769049

[CR3] Barnes L, Dumas M, Juan M et al (2010) GammaH2AX, an accurate marker that analyzes UV genotoxic effects on human keratinocytes and on human skin. Photochem Photobiol 86:933–941 20492564 10.1111/j.1751-1097.2010.00744.x

[CR4] Braselmann H, Michna A, Heß J, Unger K (2015) CFAssay: statistical analysis of the colony formation assay. Radiat Oncol 10:223. 10.1186/s13014-015-0529-y 26537797 10.1186/s13014-015-0529-y PMC4634140

[CR5] de Feraudy S, Revet I, Bezrookove V et al (2010) A minority of foci or pan-nuclear apoptotic staining of γH2AX in the S phase after UV damage contain DNA double-strand breaks. Proc Natl Acad Sci U S A 107:6870–6875. 10.1073/pnas.1002175107 20351298 10.1073/pnas.1002175107 PMC2872460

[CR6] Desouky O, Ding N, Zhou G (2015) Targeted and non-targeted effects of ionizing radiation. J Radiat Res Appl Sci 8:247–254. 10.1016/j.jrras.2015.03.003

[CR7] Dhuppar S, Roy S, Mazumder A (2020) γH2AX in the S phase after UV irradiation corresponds to DNA replication and does not report on the extent of DNA damage. Mol Cell Biol 40:e00328-20. 10.1128/MCB.00328-20 32778572 10.1128/MCB.00328-20 PMC7523655

[CR8] Diffey BL (2002) Human exposure to solar ultraviolet radiation. J Cosmet Dermatol 1:124–130 17147711 10.1046/j.1473-2165.2002.00060.x

[CR9] Firsanov DV, Solovjeva LV, Svetlova MP (2011) H2AX phosphorylation at the sites of DNA double-strand breaks in cultivated mammalian cells and tissues. Clin Epigenetics 2:283–297. 10.1007/s13148-011-0044-4 22704343 10.1007/s13148-011-0044-4 PMC3365398

[CR10] Franken NAP, Rodermond HM, Stap J et al (2006) Clonogenic assay of cells in vitro. Nat Protoc 1:2315–2319. 10.1038/nprot.2006.339 17406473 10.1038/nprot.2006.339

[CR11] Guardamagna I, Bassi E, Savio M et al (2020) A functional in vitro cell-free system for studying DNA repair in isolated nuclei. J Cell Sci 133:jcs240010. 10.1242/jcs.240010 32376788 10.1242/jcs.240010

[CR12] Guardamagna I, Lonati L, Savio M et al (2021) An integrated analysis of the response of colorectal adenocarcinoma Caco-2 cells to X-Ray exposure. Front Oncol 11:2029. 10.3389/fonc.2021.688919 10.3389/fonc.2021.688919 PMC8209426 34150657

[CR13] Hagiwara Y, Niimi A, Isono M et al (2017) 3D-structured illumination microscopy reveals clustered DNA double-strand break formation in widespread γH2AX foci after high LET heavy-ion particle radiation. Oncotarget 8:109370–109381. 10.18632/oncotarget.22679 29312614 10.18632/oncotarget.22679 PMC5752527

[CR14] Halicka HD, Huang X, Traganos F et al (2005) Histone H2AX phosphorylation after cell irradiation with UV-B: relationship to cell cycle phase and induction of apoptosis. Cell Cycle 4:339–345. 10.4161/cc.4.2.1486 15655354

[CR15] Hanasoge S, Ljungman M (2007) H2AX phosphorylation after UV irradiation is triggered by DNA repair intermediates and is mediated by the ATR kinase. Carcinogenesis 28:2298–2304. 10.1093/carcin/bgm157 17615256 10.1093/carcin/bgm157

[CR16] Hutchinson F (1985) Chemical changes induced in DNA by ionizing radiation. In: Cohn WE, Moldave K (eds) Progress in nucleic acid research and molecular biology. Academic Press, pp 115–154 10.1016/s0079-6603(08)60347-5 10.1016/s0079-6603(08)60347-5 3003798

[CR17] IARC Working group on the evaluation of carcinogenic risks to humans (2012) A review of human carcinogens. In: IARC Monographs on the Evaluation of Carcinogenic Risks to Humans. International Agency for Research on Cancer, Lyon

[CR18] Kciuk M, Marciniak B, Mojzych M, Kontek R (2020) Focus on UV-induced DNA damage and repair-disease relevance and protective strategies. Int J Mol Sci 21:7264. 10.3390/ijms21197264 33019598 10.3390/ijms21197264 PMC7582305

[CR19] Klokov D, MacPhail SM, Banáth JP et al (2006) Phosphorylated histone H2AX in relation to cell survival in tumor cells and xenografts exposed to single and fractionated doses of X-rays. Radiother Oncol 80:223–229. 10.1016/j.radonc.2006.07.026 16905207 10.1016/j.radonc.2006.07.026

[CR20] Lo HL, Nakajima S, Ma L et al (2005) Differential biologic effects of CPD and 6–4PP UV-induced DNA damage on the induction of apoptosis and cell-cycle arrest. BMC Cancer. 10.1186/1471-2407-5-135 10.1186/1471-2407-5-135 PMC1276790 16236176

[CR21] Mao Z, Bozzella M, Seluanov A, Gorbunova V (2008) DNA repair by nonhomologous end joining and homologous recombination during cell cycle in human cells. Cell Cycle 7:2902–2906. 10.4161/cc.7.18.6679 18769152 10.4161/cc.7.18.6679 PMC2754209

[CR22] Mariotti LG, Pirovano G, Savage KI et al (2013) Use of the γ-H2AX assay to investigate DNA repair dynamics following multiple radiation exposures. PLoS ONE 8:e79541. 10.1371/journal.pone.0079541 24312182 10.1371/journal.pone.0079541 PMC3843657

[CR23] Marti TM, Hefner E, Feeney L et al (2006) H2AX phosphorylation within the G1 phase after UV irradiation depends on nucleotide excision repair and not DNA double-strand breaks. Proc Natl Acad Sci U S A 103:9891–9896. 10.1073/pnas.0603779103 16788066 10.1073/pnas.0603779103 PMC1502549

[CR24] Rogakou EP, Pilch DR, Orr AH et al (1998) DNA double-stranded breaks induce histone H2AX phosphorylation on serine 139. J Biol Chem 273:5858–5868. 10.1074/jbc.273.10.5858 9488723 10.1074/jbc.273.10.5858

[CR25] Roy S (2017) Impact of UV radiation on genome stability and human health. In: Ahmad SI (ed) Ultraviolet light in human health, diseases and environment. Springer International Publishing, Cham, pp 207–219. 10.1007/978-3-319-56017-5_17

[CR26] Tanaka T, Huang X, Halicka HD et al (2007) Cytometry of ATM activation and histone H2AX phosphorylation to estimate extent of DNA damage induced by exogenous agents. Cytometry A 71A:648–661. 10.1002/cyto.a.20426 10.1002/cyto.a.20426 PMC3855668 17622968

[CR27] Ward IM, Chen J (2001) Histone H2AX is phosphorylated in an ATR-dependent manner in response to replicational stress. J Biol Chem 276:47759–47762. 10.1074/jbc.C100569200 11673449 10.1074/jbc.C100569200

[CR28] Ward IM, Minn K, Chen J (2004) UV-induced ataxia-telangiectasia-mutated and Rad3-related (ATR) activation requires replication Stress*. J Biol Chem 279:9677–9680. 10.1074/jbc.C300554200 14742437 10.1074/jbc.C300554200

