# Supplementary material for: Temporal and spatial patterns of γH2AX signaling in different human cells after exposure to X-rays and UV-C light
Source: Radiat Environ Biophys. 2026 May 22;65(2):781–91. doi: 10.1007/s00411-026-01220-z (PMC13303546; doi:10.1007/s00411-026-01220-z)
Supplement: Supplementary file 1 [file 411_2026_1220_MOESM1_ESM.pdf]

Supplementary material to:
Temporal and spatial patterns of γH2AX signaling in different human cells after exposure to X-rays and UV-C light

Isabella Guardamagna[1], Leonardo Lonati[1,*], Ombretta Iaria[1], Alice Mentana[1], Daniele Parodi[1], Giulia Peterlin[1], Rossella Semerano[1], Cecilia Riani[1], Andrea Previtali[2], Anna Tricarico[2], Paola Tabarelli de Fatis[3], Giovanni Battista Ivaldi[4], Paola Perucca[2], Ornella Cazzalini[2], Giorgio Baiocco[1]

[1]Laboratory of Radiation Biophysics and Radiobiology, A. Volta Department of Physics, University of Pavia, 27100 Pavia, Italy.

[2]Unit of Immunology and General Pathology, Department of Molecular Medicine, University of Pavia, 27100 Pavia, Italy.

[3]Unit of Medical Physics, Istituti Clinici Scientifici Maugeri IRCCS, 27100 Pavia, Italy.

[4]Unit of Radiation Oncology, Istituti Clinici Scientifici Maugeri IRCCS, 27100 Pavia, Italy.

* *corresponding author, email: leonardo.lonati@unipv.it*

γH2AX foci scoring from fluorescence microscopy images

Methods: γH2AX nuclear foci were counted for samples exposed to X-rays. Images were analyzed using Python 3.10.13. Nuclei-stained images served as input for the Cellpose model v4.0.4 (Pachitariu et al. 2025), which was used to perform nuclei segmentation. The segmentation results were subsequently fine-tuned based on the expected size range of the nuclei. Following nuclei segmentation, foci were detected using two criteria: the Difference of Gaussians (DoG) method and an area threshold between 0.2–1.5 μm^2. The DoG method highlights small bright spots by subtracting two blurred versions of the image (one slightly blurred, the other more strongly). Foci were identified by selecting the top 1% of the brightest pixels in the DoG image and then labeled for counting. Each data point was obtained as average of three independent experiments, with the error bars representing the SEM. The number of foci in non-irradiated cells (CTR) was subtracted from that of irradiated cells for each dose, obtaining the extra foci yield per cell (ΔFoci).

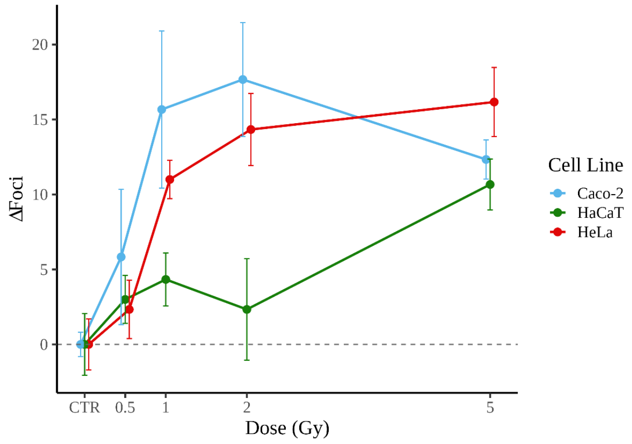

Figure S1– γH2AX foci scoring vs. X-ray dose. Data are given as extra foci yields ΔFoci per cell after subtraction of foci scored in the control (CTR) condition for Caco-2 (blue), HaCaT (green) and HeLa (red) cells exposed to different X-ray doses. Lines are drawn to guide the eye.

Results: Figure S1 presents ΔFoci per cell in HeLa (red), HaCaT (green), Caco-2 (blue) irradiated at different X-ray doses. The intrinsic difficulty in the foci quantification is reflected in the large error bars. No substantial differences in the number of ΔFoci were observed between Caco-2 and HeLa at all doses. HaCaT cells seem less responsive in terms of γH2AX foci appearance (as also found quantifying total γH2AX fluorescence), at least for the intermediate doses of 1 and 2 Gy doses, while ΔFoci rises at the same level as for the other cell lines at the highest 5 Gy dose. A saturation effect also becomes apparent starting at the 2Gy dose, at least for Caco-2 and HeLa cells.

Dose-response and kinetics of γH2AX signal after X-ray or UV-C exposure: nonlinear regression

Dose-response data for the γH2AX signal following exposures to both radiation agents were obtained with two techniques (immunofluorescence microscopy and flow cytometry), and showed two main trends: a linear increase of the fractional difference of MFI vs dose, or an increase up to signal saturation. We modelled these two behaviors applying a nonlinear regression for each cell line to a linear model (Δfrac MFI $=m\cdot d + q$) or a saturating model (Δfrac MFI $=a\cdot(1-e^{-b\cdot d})$, where d is the dose of X-rays radiation (in Gy) or the UVC exposure (in J/m^2), and *m*, *q* and *a*, *b* are free fit parameters.
Kinetics data for the γH2AX signal following exposures to both radiation agents were obtained by means of flow-cytometry, for cells irradiated with 5 Gy or 20 J/m^2 UV-C. We modelled the kinetics applying a nonlinear regression for each cell line to the following model: Δfrac MFI $=A\cdot(1-e^{-B\cdot t})\cdot e^{C\cdot t}$, where *t* is the post-exposure time, and *A*, *B* and *C* are free-parameters).
In the following tables we report for each cell line the chosen model, the optimal parameter values for dose response datasets from immunofluorescence microscopy (Table S1) and flow cytometry (Table S2), and for kinetics data (Table S3).
Each optimized parameter is reported with 95% CI, along with two figures of merit: RMSE (Root Mean Square Error) and adjusted R squared. The last column in both Tables shows the test results with an F-test, a replicate test for the lack of fit. If the model is correct, the corresponding F ratios should be close to 1 and associated to a large p-value. A small p-value is evidence that the data follows a model that is different than the chosen one (Seber and Wild, 2003).

Table S1 *Fit results for dose-response data obtained by microscopy. m, b units of measure are Gy^{-1} or m^2/J when the agent is X-rays or UV-C, respectively. q, a are in arbitrary units.*

Immunofluorescence Microscopy

Cell line	Agent	Model type	m (95% CI)	q (95% CI)	a (95% CI)	b (95% CI)	RMSE	adj R²	F ratio, p-value
HeLa	X-rays	Saturating	-	-	0.75 (0.51–2.67)	1.32 (0.60–7.06)	0.36	0.21	F 2.088 p 0.107
Caco-2	X-rays	Saturating	-	-	0.79 (0.59–1.03)	1.32 (0.60–7.05)	0.53	0.31	F 1.018 p 0.388
HaCaT	X-rays	Linear	0.042 (0.008–0.076)	-0.129 (-0.207–-0.052)	-	-	0.60	0.29	F 2.677 p 0.052

HeLa	UV-C	Saturating	-	-	1.46 (1.21–1.80)	0.14 (0.07–0.42)	0.73	0.35	F 0.481 p 0.619
Caco-2	UV-C	Saturating	-	-	2.58 (1.37–9.85)	0.026 (0.002–0.087)	0.34	0.81	F 0.980 p 0.380
HaCaT	UV-C	Linear	0.0074 (0.0023–0.0125)	0.1763 (0.0636–0.2890)	-	-	0.84	0.33	F 2.782 p 0.051

Table S2 *Fit results for dose-response data obtained by flow-cytometry. m, b units of measure are Gy^{-1} or m^2/J when the agent is X-rays or UV-C, respectively. q, a are in arbitrary units.*

**Cell line**	***Agent***	***Model type***	***m* (95% CI)**	***q* (95% CI)**	***a* (95% CI)**	***b* (95% CI)**	***RMSE***	***adj R^2***	***F ratio, p-value***
HeLa	X-rays	Saturating	-	-	3.66 (1.65–8.09)	0.145 (0.041–0.496)	0.27	0.81	F 0.287 p 0.833
Caco-2	X-rays	Saturating	-	-	1.29 (0.97–1.93)	0.617 (0.26–1.31)	0.20	0.79	F 0.909 p 0.471
HaCaT	X-rays	Linear	0.070 (0.036–0.103)	-0.017 (-0.100–0.065)	-	-	0.10	0.58	F 0.250 p 0.860
HeLa	UV-C	Saturating	-	-	2.15 (1.71–2.93)	0.092 (0.042–0.226)	0.32	0.86	F 0.001 p 0.998
Caco-2	UV-C	Linear	0.042 (0.032–0.051)	-0.015 (-0.230–0.200)	-	-	0.21	0.90	F 3.041 p 0.102
HaCaT	UV-C	Linear	0.024 (0.008–0.039)	0.098 (-0.206–0.457)	-	-	0.34	0.49	F 0.634 p 0.555

Table S3 *Fit results for kinetics data obtained by flow-cytometry. B, C units of measure are h^{-1}, A is in arbitrary units.*

**Cell line**	**Agent**	**A (95% CI)**	**B (h^{-1}; 95% CI)**	**C (h^{-1}; 95% CI)**	**RMSE**	**adj R^2**	**F ratio. p-value**
HeLa	X-rays	4.331 (3.276–6.898)	5.237 (1.639–12.329)	0.087 (0.028–0.238)	1.33	0.54	F 0.940 p 0.491
Caco-2	X-rays	3.778 (3.235–4.515)	9.432 (4.499–18.732)	0.111 (3.235–4.515)	0.67	0.80	F 16.98 p 0.001
HaCaT	X-rays	2.106 (1.515–3.444)	0.813 (-0.361–1.635)	0.044 (0.016–0.097)	0.36	0.69	F 0.573 p 0.747
HeLa	UV-C	7.272 (6.277–8.464)	1.112 (0.802–1.547)	0.015 (0.003–0.028)	0.89	0.85	F 0.644 p 0.694
Caco-2	UV-C	3.218 (2.788–	0.858 (0.623–	-0.013 (-0.017–	0.48	0.93	F 6.719 p 0.003

		3.698)	1.189)	-0.009)			
HaCaT	UV-C	2.037 (1.505–2.688)	1.738 (0.807–5.690)	-0.034 (-0.049–-0.018)	0.72	0.74	F 0.289 p 0.935

Cell-Cycle-Resolved γH2AX Signaling

Methods: HaCaT, Caco-2 and HeLa cells were seeded at 500 000 in 65 mm Petri dishes (for UVC irradiation) and in T25 flasks for X-rays irradiation. 24 h after seeding, samples were irradiated with the two types of radiation (5 Gy of X-rays and 20 J/m^2 for UV-C) as described in Materials and methods section of the main manuscript. Cells were then incubated at 37°C in a humidified atmosphere with 5% CO_2. At defined time points (20', 40' and 1, 2, 4, 6 and 24 hours) cells were then collected by trypsinization and fixed with 4% paraformaldehyde for 5 min and permeabilized with 70% ethanol in 0.9% NaCl. Samples were incubated in blocking solution (PBS, 0.2% Tween-20 containing 5% BSA) and subsequently incubated with the anti-γ-H2AX primary antibody (1:100 dilution, Cell Signaling Technology, RRID: AB_1640564) for 1h and then, with the secondary anti-rabbit antibody (1:200 dilution, 488, RRID:AB_10679405) for 30 min. DNA staining was obtained using the FxCycle™ Violet Stain kit (Invitrogen™ ThermoFisher Scientific). At least 3 biological replicates for each cell line were acquired using an Attune NxT Acoustic Focusing flow cytometer (ThermoFisher Scientific). All the collected data were analysed using the Attune NxT software v 4.2.1627.1.

Distributions spectra of γH2AX and FxCycle Violet signals where gated according to the cell DNA content, subdividing populations in G1, S and G2M -phases. We report the kinetics of the fractional difference signal (Δfrac MFI = (MFI $_{condition}$ – MFI $_{control}$)/ MFI $_{control}$, with respect to the average controls, evaluated at 6 and 24 h) of the MFI signal of γH2AX in each phase, for cells exposed to both types of radiation.

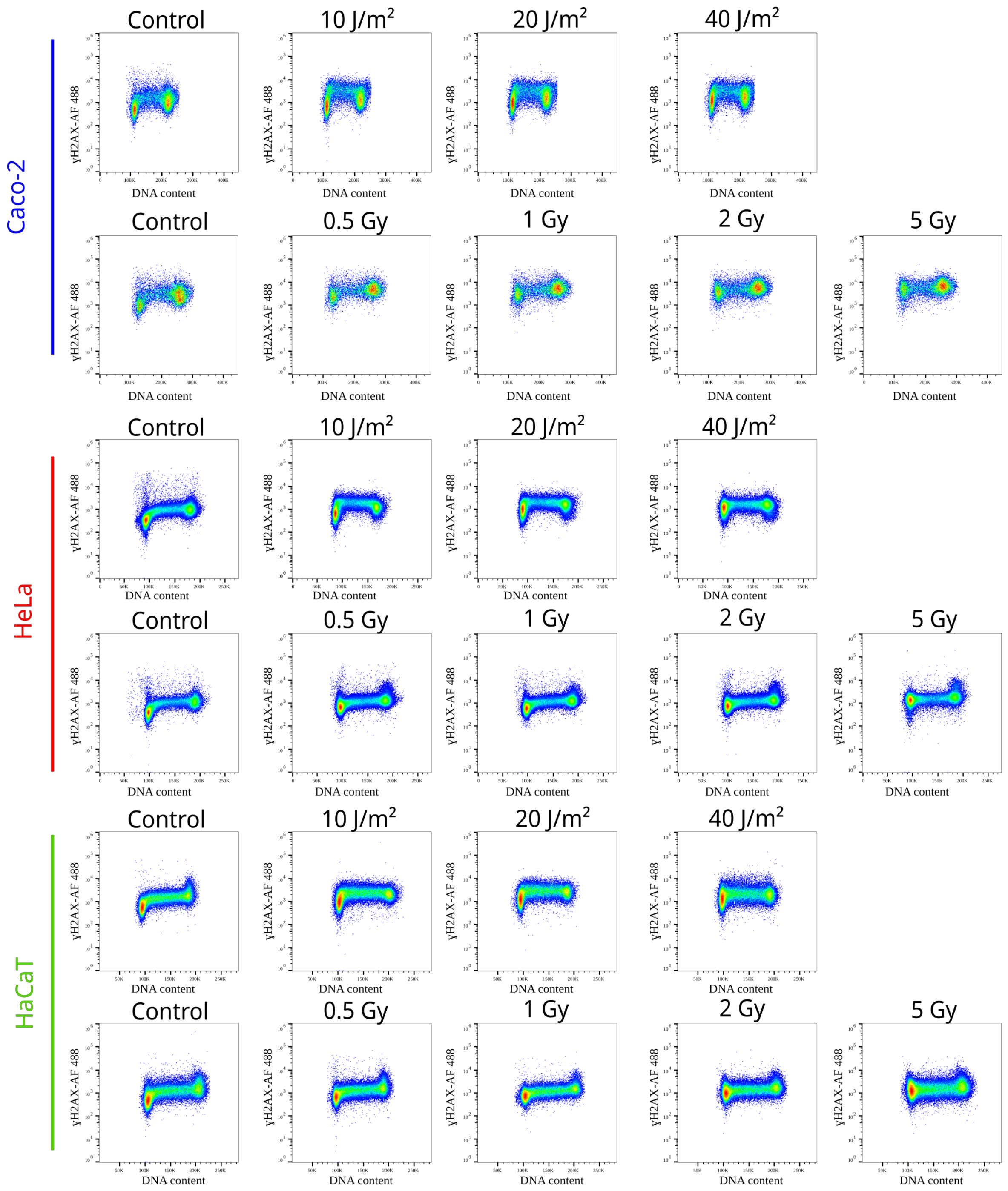

Figure S2 – Representative flow cytometry biparametric plots for γH2AX fluorescence signal vs DNA content for each cell line (Caco-2, in blue, HeLa in red and HaCaT in green) and doses, (UV-C and X-rays.

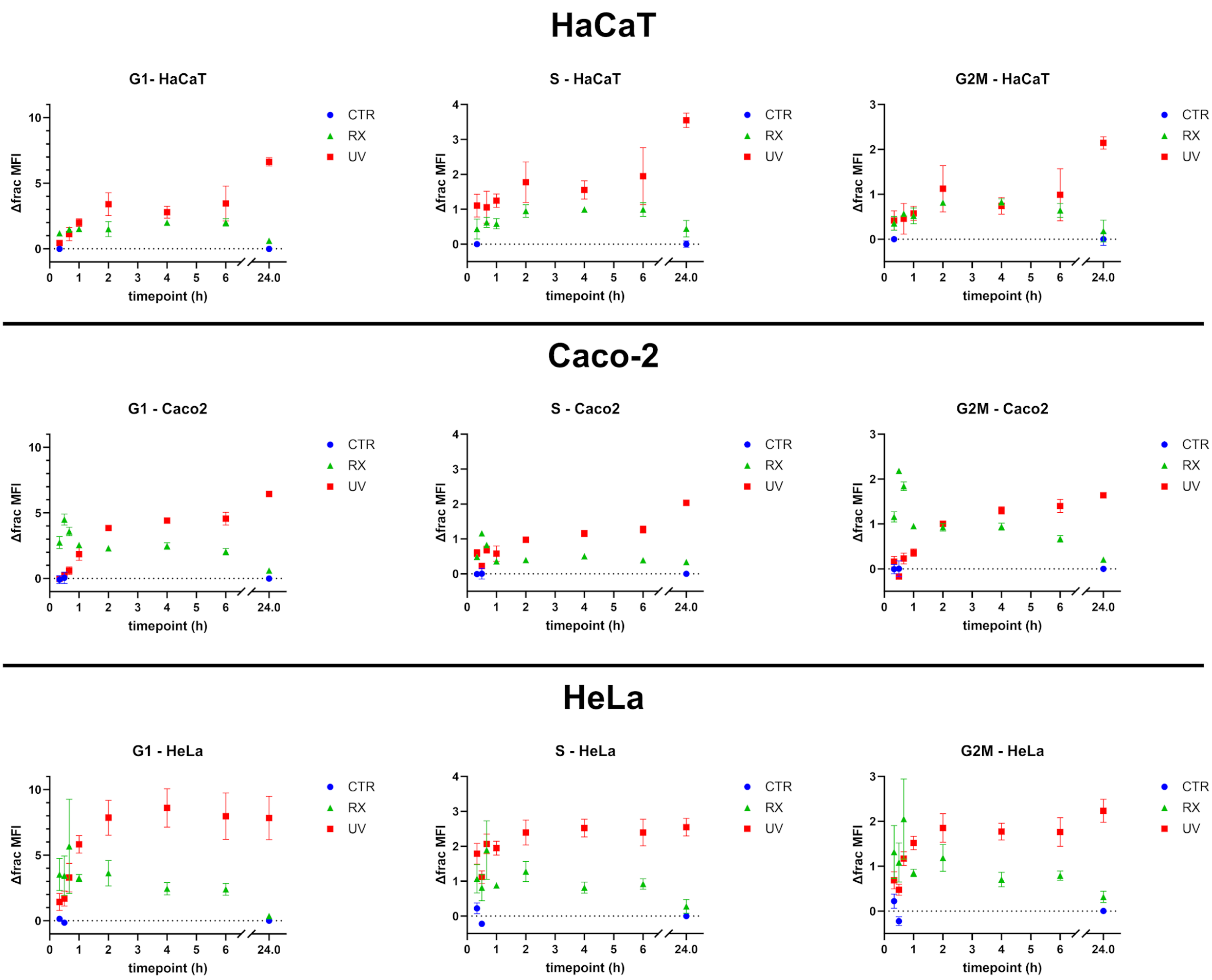

Figure S3 – Cell-cycle phase resolved data expressed as Δfrac MFI of the γH2AX signal kinetics after X-ray (5 Gy) and UV-C (20 J/m^2) exposures from flow-cytometry analysis.

Results: Figure S3 shows the Δfrac MFI of γH2AX for each cell line and for each phase as a function of time after exposure to 5 Gy of X-rays and 20 J/m^2 for UV-C. Control samples (CTR) did not show any significant difference in the signal for cell-cycle phases at different time points. After UVC exposure, the γH2AX signal of cells in the S-phase seems amplified already at earlier times (<1h) after irradiation, but any difference with signals from cells in other phases disappears quickly, and the overall trends of Δfrac MFI vs. time seem to not be phase-dependent. The same conclusion holds for X-ray irradiations. The Δfrac MFI of γH2AX from cells in G1-phase is always ~4 times higher than that in the other phases, for both types of radiation: this could be related to the higher abundance of cells in G1-phase, dominating the amplification of the signaling.

Clonogenic survival after UV-C exposure

Methods: Cells were seeded in 60mm cell culture dishes at different densities as specified in **Table S1**. After two days, cells were exposed to 5-10-20-40 J/m^2 UV-C radiations, immediately trypsinized and harvested and re-seeded in 60mm cell culture dishes. The plating efficiency (PE) was assessed with cells that were not exposed to UV-C radiations. After 7–10 days the colonies were washed twice in PBS and stained with Gentian Violet for 20 min under constant stirring. Then,

the dye was washed several times with distilled water and the colonies were air dried and counted. Colonies were counted with a colony counter (SC6Plus, Stuart, Cernusco sul Naviglio, Italy), the Survival Fraction was scored as the ratio between the number of colonies counted divided by the number of seeded cells, normalized by the PE.

Table S4: Number of cell seeded for the colony forming assay for each cell line and for each exposure level of UV-C treatment.

**Cell line**	**Control**	**5 J/m^2**	**10 J/m^2**	**20 J/m^2**	**40 J/m^2**
HeLa	200	500	10 000	20 000	20 000
Caco-2	500	20 000	40 000	40 000	40 000
HaCaT	1 500	60 000	120 000	120 000	120 000

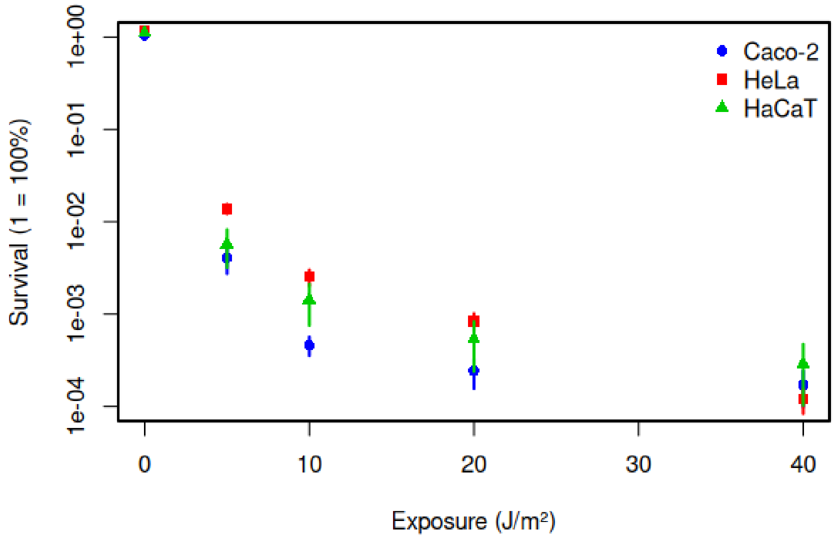

Figure S4 – Survival fraction for Caco-2 cells (blue dots), HeLa cells (red squares), and HaCaT cells (green triangles), after irradiation with different exposure levels of UVC. Data are expressed as means ± SEM of at 3 replicates.

Results: UV-C treatment led to a strong dose-dependent reduction in colony-forming ability across all cell lines (**Figure S4**). These results indicate that UV-C exposure substantially impairs the proliferative capacity of all three cell lines, consistent with the persistence of γH2AX signal observed in flow-cytometry analyses. Differences in the relative radiosensitivity to UVC among the cell lines can be inferred only at the lower (< 20 J/m^2) exposure levels.

Bibliography

Marius Pachitariu (2025) Cellpose-SAM: superhuman generalization for cellular segmentation. https://doi.org/10.1101/2025.04.28.651001

Seber and Wild (2003) *Nonlinear Regression,* CAF Seber and CJ Wild, Wiley Interscience, 2003, pages 30-32. https://doi.org/10.1002/0471725315
